# Character pathology and neuropsychological test performance in remitted opiate dependence

**DOI:** 10.1186/1747-597X-3-23

**Published:** 2008-11-19

**Authors:** James M Prosser, Daniel Eisenberg, Emily E Davey, Matthew Steinfeld, Lisa J Cohen, Edythe D London, Igor I Galynker

**Affiliations:** 1 Department of Psychiatry and Behavioral Sciences, Beth Israel Medical Center, First Avenue at 16th St, New York, NY 10003, USA; 2 National Institute of Mental Health, Division of Clinical Research. Bldg. 10, Magnusen CC, 10 Center Dr. Bethesda, MD, USA; 3 Department of Psychology at the New School for Social Research, New York, NY, USA; 4 Department of Medicine and Molecular Pharmacology, Brain Research Institute, David Geffen School of Medicine, University of California at Los Angeles, USA; 5 Department of Psychiatry, Beth Israel Medical Center, First Avenue at 16th St, New York, NY 10003, USA

## Abstract

**Background:**

Cognitive deficits and personality pathology are prevalent in opiate dependence, even during periods of remission, and likely contribute to relapse. Understanding the relationship between the two in vulnerable, opiate-addicted patients may contribute to the design of better treatment and relapse prevention strategies.

**Methods:**

The Millon Multiaxial Clinical Inventory (MCMI) and a series of neuropsychological tests were administered to three subject groups: 29 subjects receiving methadone maintenance treatment (MM), 27 subjects in protracted abstinence from methadone maintenance treatment (PA), and 29 healthy non-dependent comparison subjects. Relationships between MCMI scores, neuropsychological test results, and measures of substance use and treatment were examined using bivariate correlation and regression analysis.

**Results:**

MCMI scores were greater in subjects with a history of opiate dependence than in comparison subjects. A significant negative correlation between MCMI scores and neuropsychological test performance was identified in all subjects. MCMI scores were stronger predictors of neuropsychological test performance than measures of drug use.

**Conclusion:**

Formerly methadone-treated opiate dependent individuals in protracted opiate abstinence demonstrate a strong relationship between personality pathology and cognitive deficits. The cause of these deficits is unclear and most likely multi-factorial. This finding may be important in understanding and interpreting neuropsychological testing deficiencies in opiate-dependent subjects.

## Background

Cognitive impairment is prevalent in opiate dependence, even during periods of remission, and may be associated with poor addiction treatment outcomes [1-7]. However, the etiology of this cognitive impairment is unclear [8,9]. Indeed, to date there is no evidence of neuronal cytopathology due to the direct effects of opiates in humans [2,10]. We have recently reported that individuals with remitted opiate dependence showed impairment on tasks measuring verbal function, visuospatial ability and memory, and resistance to distractibility, which was unrelated to measures of drug abuse and methadone history but varied to a small degree with treatment status (agonist treatment with methadone versus opiate abstinent treatment) [8]. Ersche et al. [9] published similar findings, in which opiate users demonstrated pronounced impairment in executive and memory functions, even when controlling for variations in opiate exposure history. These findings have lead several authors to hypothesize that cognitive impairment in opiate dependence may not entirely result from direct opiate effects on the brain, but rather may be related to other factors associated with vulnerability to addiction [8-13].

Because personality disorders are the most prevalent comorbid psychiatric condition in opiate dependence [14], and are associated with cognitive dysfunction in non-addicted populations [15-20], it is important to investigate the relationship between personality disorders and neuropsychological functioning in opiate addiction.

Several reports have used the Millon Clinical Multiaxial Inventory (MCMI) to document increased prevalence of particular types of personality pathology in opiate dependence [21-25]. Craig [22] documented the prominence of antisocial (60%), passive-aggressive (34%), and depressive (32%) personality disorders in opiate addicted subjects. More recently, Teplin et al. [25] found that 77% of patients in a methadone maintenance clinic met criteria for at least one personality disorder, with depressive being most common (31%), followed by dependent (26%) and masochistic (20%). Cohen et al. [23] confirmed that former opiate abusers had notably higher scores on clusters A, B, and C MCMI scales when compared to controls, but also found greater cluster A pathology in subjects withdrawn from methadone compared to methadone maintained subjects. Additionally, personality pathology identified in opiate dependence has been shown to be significantly correlated with adverse outcomes related to employment, family relationships, psychiatric health, HIV risk, social judgment, and relapse [26,27]. However, none of these studies examined the relationship between personality pathology and neuropsychological functioning.

In fact, to date, we are aware of no research on the relationship of personality pathology and neuropsychological functioning in opiate-addicted individuals, though an association between neuropsychological deficits and personality pathology, unrelated to substance abuse, has been documented [17,18,20,28].

Thus, because both cognitive impairment and personality disorders are prevalent and associated with poor treatment outcomes in opiate addicted individuals, understanding the relationship between these pathologies may broaden our understanding of both impairments in these patients, as well as aid in designing more effective addiction treatments. We therefore sought to examine this relationship in two groups of treated opiate dependent individuals: those receiving methadone maintenance and those previously maintained with methadone and currently in protracted opiate abstinence. The subjects were tested using well-accepted measures of neuropsychological performance and personality pathology, and the relationships of these variables were examined using correlation and linear regression. We hypothesize that 1) personality pathology will correlate with neuropsychological test performance; 2) that the effect of drug use on neuropsychological test performance will be greater than the effect of personality pathology on neurological test performance; and 3) these relationships will be similar for both subjects currently receiving methadone and subjects who are opiate-abstinent.

## Methods

### Subjects

Eighty-five subjects (64 male, 21 female, 21–55 years of age) were selected for inclusion in one of three groups based on their drug use history: 1) Opiate-dependent subjects receiving methadone-maintenance therapy (MM); 2) Opiate dependent subjects previously detoxified from methadone maintenance therapy and in a state of protracted opiate abstinence (PA); and 3) comparison subjects without a history of opiate-dependence (C) (Table 1). For inclusion in the MM or PA groups, participants were required to meet the DSM-IV criteria for opiate dependence in the 2 years prior to the study and to have been free of illicit drug use for the previous 18 months. Furthermore, both MM and PA subjects were required to have negative urine toxicology screening tests (excluding methadone in the MM group) as reported by their respective treatment programs. The MM subjects were recruited from either the Su Casa Short Stay Residence (7 Gouverneur Slip East, New York, NY 10002), or the Beth Israel Medical Center Methadone Maintenance treatment program. The inclusion criteria for the MM group consisted of enrollment in the methadone maintenance program for at least one year and a stable methadone dose for the previous 6 months. The PA subjects were recruited from the Su Casa Methadone to Abstinence Residence (7 Gouverneur Slip East, New York, NY 10002). The inclusion criterion for the PA group was prior methadone maintenance for at least 1 year, followed by gradual methadone detoxification to complete abstinence, and no methadone treatment in the 6 months prior to testing. Non-dependent comparison subjects were recruited through advertising in a local weekly magazine.

**Table 1:** Demographic data and substance use history of methadone maintained, protracted abstinence and non-dependent comparison groups, using Chi-squared testing (χ^2^) for categorical variables, and ANOVA (F) for continuous variables.

	**Methadone Maintained** (n = 29)	**Protracted Abstinence** (n = 27)	**Comparison** (n = 28)	**Statistics**	**Significance**
**Gender**					
Male	23 (79.3%)	20 (74%)	21(72.4%)	χ^2 ^= 0.49	*p *= 0.78
Female	6 (20.7%)	7 (26%)	8 (27.6%)	df = 2	

**Ethnicity**					
African American	6 (20.7%)	11 (40.7%)	10 (34.7%)		
European American	11 (37.9%)	7 (25.9%)	12 (41.4%)		
Hispanic	12 (41.4%)	7 (25.9%)	3 (10.3%)	χ^2 ^= 14.95 df = 10	*p *= 0.13
Asian	0 (0%)	0 (0%)	1 (3.4%)		
Native Am./Pacific Islands	0 (0%)	0 (0%)	1 (3.4%)		
Other	0 (0%)	2 (7.5%)	2 (6.8%)		

**Age **Years: Mean ± S.D.	37.93 ± 7.5	42.59 ± 5.4	34.00 ± 8.0	F(2,84) = 10.21	p < 0.001

**Education **Years: Mean ± S.D.	13.03 ± 4.8	11.81 ± 2.2	15.51 ± 1.8	F(2,84) = 9.32	p < 0.001

**Substance Use History**					
Mean ± S.D.					
Age of Onset: Years	19.36 ± 5.5	23.32 ± 8.9	N/A	F = 3.88; df = 1	*p *= 0.054
Years of heroin use	14.72 ± 8.9	14.68 ± 9.0	N/A	F = 0.00 df = 1	*p *= 0.98
Daily dollar amount: $	122.96 ± 105.09	124.57 ± 115.90	N/A	F = 0.003 df = 1	*p *= 0.959
**Substance Treatment History**					
Mean ± S.D.					
Years of MM	6.48 ± 7.03	6.85 ± 6.80	N/A	F = 0.038 df = 1	*p *= 0.847
Highest methadone dose (mg/day)	88.21 ± 25.54	83.68 ± 30.22	N/A	F = 0.307 df = 1	*p *= 0.582
Years of opiate abstinence	N/A	0.89 ± 0.37	N/A	N/A	

N/A = not applicable

Years of MM = years of methadone maintenance

Exclusion criteria for all participants were: current or lifetime history of any Axis I diagnosis (other than opiate dependence for the MM and PA groups, and nicotine dependence for all groups), current alcoholic intake of more than 15 drinks (1.5 oz. liquor, 12 oz. beer, or 5 oz. wine equivalents) per week, history of head trauma, cardiovascular, pulmonary, other systemic, or neurological disease, and HIV seropositivity. Neither moderate use of caffeine, (<600 mg of caffeine per day), nor occasional marijuana use (≤ 1 marijuana cigarette/month and urine toxicology negative for THC) were exclusionary.

Initial screening was performed in a telephone interview. Subjects meeting initial inclusion/exclusion criteria and who continued to express interest in participating were invited for a face-to-face interview, consisting of the Structured Clinical Interview for DSM (SCID-I), conducted by trained personnel [29]. All subjects received a physical examination, routine laboratory testing, and a "surprise" urine drug-screening exam. A subset of subjects who participated received two additional urine drug-screening tests. Su Casa counselors also confirmed that subjects from their facility had negative urine drug tests. All interested subjects who met inclusion and exclusion criteria were entered into the study after signing a consent form. In all, 29 subjects were enrolled in the MM group, 27 subjects in the PA group, and 28 subjects in the non-dependent comparison group. All subjects received a small financial reimbursement for their participation. All procedures were reviewed and approved by the Institutional Review Board of Beth Israel Medical Center.

### Outcome measures

Cognitive performance was determined by a small battery of tests, designed to assess aspects of cognitive functioning most frequently cited as disrupted in the addiction literature, assess neuropsychological function in multiple domains, and be quick and efficient. These were:

• *The WAIS-R Vocabulary Test *[30]: A test of verbal function and an estimate of general IQ. This is the WAIS-R subtest most highly correlated with full scale IQ. The WAIS-R was used instead of the WAIS-III to be consistent with earlier data collection. Age-corrected standard scores are derived from the raw subtest data, and have an average of 10, and a standard deviation of 3.

• *The Stroop Color-Word Test *[31,32]: A test of sustained attention and resistance to distraction. The subjects are asked to name the printed color of displayed words. Only Stroop Color-Word (CW) and Stroop Interference scores were used. The number of correct responses in the allotted time determines the CW score (raw score), while the Interference score is derived mathematically (Interference = CW – age/education predicted score).

• *The Controlled Oral Word Association (COWA) *[33-35]: A test of verbal fluency. Subjects are asked to name as many words possible beginning with the letters C, F, or L in a sixty-second period. The total numbers of correct responses were recorded.

• *The Benton Visual Retention Test (BVRT): *A test of visual memory, and visual construction. Subjects are presented an image for 10 seconds and then asked to draw it from memory. The numbers of total correct, total errors, right, and left-sided errors are recorded [36-38]. Only BVRT Correct scores were analyzed.

Substance use history was determined by:

• *Substance Use Inventory (SUI) *[39]: A questionnaire about quantity and frequency of use of abused substances (opiates, cocaine, alcohol, marijuana, amphetamines, sedatives, PCP, and prescription medications). MM and PA respondents reported measures related to substance use (age of first heroin use, years of heroin abuse, approximate dollars spend daily on heroin) and substance treatment (years of methadone use, highest methadone dose), and PA subjects only reported on years since methadone detoxification). Non-dependent comparison subjects were asked to respond regarding the last 30 days and former opiate abusers were asked to refer to lifetime use.

Personality pathology was measured by:

• *Millon Clinical Multiaxial Inventory-II (MCMI) *[40]: This 175-item questionnaire measures personality disorders as well as several Axis I disorders and syndromes (as defined in DSM-III-R). Seventeen scale scores were calculated according to the scoring key in the MCMI-II manual. No corrections were used in scoring. Following the definitions of the American Psychiatric Association [41], we assigned scores for the three cluster sub-types: cluster A – (paranoid, schizoid, and schizotypal personality disorders), cluster B (antisocial, borderline, histrionic, and narcissistic personality disorders), and Cluster C (avoidant, dependent, and obsessive-compulsive personality disorders). Total scores for clusters A, B and C, were calculated, and a composite MCMI score constructed by adding together the cluster scores.

### Statistical analysis

Initial analyses compared groups on demographic and clinical measures (e.g. years of heroin abuse) with ANOVA's for continuous variables and Chi-Square analyses for categorical variables.

The scores for personality pathology (MCMI Composite score, MCMI Cluster A score, MCMI Cluster B score, and MCMI Cluster C score) were compared across groups using MANOVA followed by Tukey's HSD pair-wise comparisons. The relationship between the MCMI scores and neuropsychological test performance were examined using bivariate correlation and regression. In order to reduce the number of multiple comparisons, the scores for the WAIS-vocabulary, COWA, Stroop CW, and Benton Correct tests were transformed to Z-scores and then combined to form a normalized composite neuropsychological performance score. The COWA total correct score was age- and education-corrected prior to normalization according to the methods of Gladsjo et al [42]. This normalized composite neuropsychological performance score was then correlated against the MCMI scores by calculating Pearson's *r*. Methadone-maintained subjects, PA subjects, and non-dependent comparison subjects were examined individually.

In order to try and control for any confounding effect of drug use history on neuropsychological test performance, we used multiple regression to regress the composite neuropsychological performance scores on combinations of the MCMI composite score and drug use history variables. Because the non-dependent comparison group did not have similar drug use history data, the regression analysis was carried out for the MM and PA groups only. Scatterplots of the variables composite neuropsychological performance score, composite MCMI score, and drug use history were constructed to confirm a linear relationship between the variables (not shown). The associations between independent and dependent variables are presented by means of standardized linear regression coefficients (β).

## Results

In all, there were 29 Methadone Maintained (MM), 27 Protracted Abstinent (PA), and 28 non-dependent comparison subjects who completed all evaluations. The three subject groups did not differ with respect to gender or ethnicity by χ^2 ^analysis. Significant group differences were identified for the variables age and education: by Tukey's HSD paired comparisons, PA subjects were older than both non-dependent comparison and MM subjects, and the non-dependent comparison subjects had more years of education than both of the drug dependent groups. Therefore, age and years of education were entered as covariates in the MANCOVA comparing neuropsychological and personality data across groups.

The MM and PA groups did not differ significantly on any measure of substance use history (age of first heroin use, duration of heroin use, approximate amount spent daily on heroin) or substance treatment history (years of methadone use, and highest dose of methadone). The difference between the MM and PA groups on mean age of first opiate use approached, but did not achieve significance. Protracted abstinence subjects were opiate-free an average of 10.73 months, with a range of 6–24 months. The demographic data is presented in Table 1. Complete details of the neuropsychological testing have been previously published [8]. Briefly, both groups of former opiate-dependent subjects performed worse than the control subjects on the WAIS-R Vocabulary subtest, the Stroop Interference score, and the BVRT Errors subscore. Additionally, PA subjects performed worse than controls subjects on the BVRT Total Correct subscore, and BVRT Left and Right Errors subscores. Subjects in protracted abstinence also performed worse than MMT subjects on BVRT total correct subscore.

By MANOVA, there was a significant overall difference across groups in MCMI results (Wilk's λ = .632 (Multivariate F = 4.99); df = 6, 116; *p *< 0.001). With age and years of education entered as covariates, this difference remained significant (Wilk's λ = .754 (Multivariate F = 2.78); df = 6, 110; *p *< 0.015). The results of the univariate F tests with age and years of education entered as covariates demonstrated significant differences in group means for the composite MCMI score, the MCMI Cluster B score, and Cluster C scores. The difference across group means of MCMI Cluster A score was marginally significant (See Table 2). By Tukey's HSD pairwise comparisons, both the MM and PA groups scored higher than the non-dependent comparison group on the composite MCMI score, the Cluster B score, and the Cluster C score. There was no statistical difference between the MM and PA groups on any of the MCMI variable scores.

**Table 2:** Mean MCMI scores for methadone maintained, protracted abstinence, and non-dependent comparison groups.

	**MM **mean (SD)	**PA **mean (SD)	**Comparison **mean (SD)	**Statistics**	Paired Comparisons*
MANCOVA				Multivariate Test: Wilk's λ (6, 110) = .754; *p *< 0.015	
Composite MCMI	385.22 (136.66)	395.68 (123.02)	243.74 (63.96)	Univariate F = 5.11, df = 2, *p *< 0.009	**A, B**
Cluster A	67.89 (32.27)	71.41 (33.85)	39.52 (18.9)	Univariate F = 2.58, df = 2, *p *= 0.084	
Cluster B	179.39 (63.7)	187.78 (55.64)	116.43 (33.42)	Univariate F = 6.19, df = 2, *p *= 0.004	**A, B**
Cluster C	137.94 (45.94)	136.50 (40.16)	87.78 (21.75)	Univariate F = 4.28, df = 2, *p *= 0.019	**A, B**

* Tukey's HSD Pairwise Comparisons: A = p < 0.05 for MM vs. Comparison, B = p < 0.05 for PA vs. Comparison, C = p < 0.05 for MM vs. PA.

MM = Methadone Maintained, PA = Protracted Abstinence.

When examining all subjects together, there was a significant negative correlation of total MCMI scores and the normalized composite neuropsychological performance score (r = -0.572, df = 82, *p *< 0.001). Similar results were observed when correlating the three MCMI cluster scores with the normalized composite neuropsychological score. This negative correlation was also evident when examining PA subjects only (r = -0.537, df = 25, p = 0.012). When analyzing either the non-dependent comparison group or the MM groups alone, the correlation of MCMI scores and the normalized composite neuropsychological scores was not statistically significant (See Table 3).

**Table 3:** Bivariate correlation of MCMI scores and normalized composite neuropsychological test performance score by group

	**ALL**	**MM**	**PA**	**Comparison**
	df = 82	df = 27	df = 25	df = 26
Composite MCMI	-0.572	-.292	-.537	-.322
	*p *< 0.001	*p *= 0.272	*p *= 0.012	*p *= 0.207
Cluster A	-0.561	-.363	-.533	-.191
	*p *< 0.001	*p *= 0.272	*p *= 0.013	*p *= 0.462
Cluster B	-0.499	-.229	-.411	-.273
	*p *< 0.001	*p *= 0.393	*p *= 0.064	*p *= 0.289
Cluster C	-0.601	-.292	-.63	-.36
	*p *< 0.001	*p *= 0.272	*p *= 0.002	*p *= 0.156

MM – Methadone Maintained, PA – Protracted Abstinence, MCMI – Millon Clinical Multiaxial Inventory.

We used linear regression to examine the functional relationship of MCMI cluster scores and drug use history variables to the composite neuropsychological performance score. When all subjects were included in the analysis, the regression of the composite MCMI cluster score on the composite neuropsychological performance score was significant (R = -0.572; *t *= 5.03, *p *< 0.001). The addition of any of the variables of drug use history (age of onset of drug use, years of heroin addiction, average dollars spent per day on heroin, years of methadone treatment, and highest dose of methadone) to the model resulted in a decrement of the coefficient of determination (r^2^) for each model tested. In all models tested, the β score for the composite MCMI score was greater than the β score of any of the variables of drug use history. In fact, none of the drug abuse variables were significantly related to the composite neuropsychological performance score (See Table 4).

**Table 4:** Multiple regression testing the effects of predictor variables on the normalized composite neuropsychological performance score.

**Subjects**	**Predictor**	**Standardized Coefficient**	**R^2^**	**ANOVA**
All	Composite MCMI	β = -0.572;	0.327	F = 25.312
		t = -5.031;		df = 1, 52
		*p *< 0.001		*p *< 0.001

MM + PA	Composite MCMI	β = -0.429;	0.185	F = 3.52;
		t = -2.65;		df = 2, 31;
		*p *= 0.013		*p *= 0.042
			
	Age at first drug use	β = -0.01;		
		t = -0.061;		
		*p *= 0.952		

MM + PA	Composite MCMI	β = -0.434;	0.188	F = 3.94;
		t = -2.8;		df = 2, 34;
		*p *= 0.008		*p *= 0.029
			
	Years of heroin addiction	β = 0.003;		
		t = 0.022;		
		*p *= 0.982		

MM + PA	Composite MCMI	β = -0.398;	0.163	F = 0.2.82;
		t = -2.34;		df = 2, 29;
		*p *= 0.026		*p *= 0.076
			
	Estimated dollars spent daily on heroin	β = 0.085;		
		t = 0.50;		
		*p *= 0.622		

MM + PA	Composite MCMI	β = -0.437;	0.195	F = 4.12;
		t = -2.84;		df = 2, 34;
		*p *= 0.008		*p *= 0.025
			
	Years of MMT	β = -0.083;		
		t = -0.536;		
		*p *= 0.596		

MM + PA	Composite MCMI	β = -0.344;	0.196	F = 3.41;
		t = -1.97;		df = 2, 28;
		*p *= 0.058		*p *= 0.047
			
	Highest dose of methadone	β = 0.21;		
		t = 1.21;		
		*p *= 0.238		

MM – Methadone Maintained, PA – Protracted Abstinence, MCMI – Millon Clinical Multi-axial Inventory.

Since the correlation between MCMI scores and neuropsychological performance was present only in the PA group, we performed a secondary analysis of the correlation of MCMI Cluster scores with the specific neuropsychological tests WAIS-R Vocabulary subscores, COWA Total subscore, Stroop CW subscore, and BVRT Total Correct subscore for the PA subjects only (Table 5). The results are presented in Table 5. A significant negative correlation of MCMI Cluster A scores were identified for the WAIS-R Vocabulary subscores. Significant negative correlations of MCMI Cluster B scores were identified for the WAIS-R Vocabulary subscores, and the Stroop CW subscores. Significant negative correlations of MCMI Cluster C scores were identified for the WAIS-R Vocabulary subscores, the Stroop CW subscores, and the BVRT Total Correct subscores.

**Table 5:** Correlation of MCMI Cluster scores and specific neuropsychological test scores for former opiate addicts in protracted abstinence.

	**Cluster A**	**Cluster B**	**Cluster C**
**WAIS-R Vocabulary**	-0.599	-0.474	-0.682
	*p *= 0.004	*p *= 0.030	*p *= 0.001

**COWA Total**	-0.347	-0.176	-0.422
	*p *= 0.123	*p *= 0.446	*p *= 0.057

**Stroop CW**	-0.373	-0.427	-0.428
	*p *= 0.087	*p *= 0.048	*p *= 0.047

**BVRT Total Correct**	-0.394	-0.263	-0.503
	*p *= 0.077	*p *= 0.248	*p *= 0.020

WAIS-R Vocabulary – Weschler Adult Intelligence Scale-Revise Vocabulary subscore, COWA Total – California Oral Word Association total correct score, Stroop CW – Stroop Color-Word subscore, BVRT Total Correct – Benton Visual Retention Test total correct subscore.

## Discussion

In agreement with our first hypothesis, we observed a significant correlation of MCMI scores and impaired neuropsychological test performance in a group of subjects with a history of opiate-dependence treated with methadone. Contrary to our second hypothesis, the regression models indicate that personality pathology explains a greater amount of the variance in neuropsychological test performance than do any of the variables of drug use history. Lastly, the relationship of personality pathology and neuropsychological performance is significant for the abstinent former opiate addicts, but not for the methadone-maintained group. We suggest that these results are best explained by postulating a multi-factorial origin to the neuropsychological deficits associated with opiate dependence, as opposed to thinking that drug use alone causes such deficits. To our knowledge, this report is the first to identify a link between personality pathology and the measurement of neuropsychological impairment in opiate addiction.

Our findings are consistent with previous studies that demonstrated significantly increased MCMI scores in opiate-dependent subjects compared to healthy controls [21-25]. Additionally, our results are consistent with those studies of non-addicted subjects that showed significantly worse neuropsychological test performance in individuals with personality disorders [16-18,20,28]. We know of no previous study that has attempted to measure the correlation of MCMI scores and neuropsychological test performance. In every instance where we measured this association, we found a negative correlation of MCMI scores and neuropsychological test performance, indicating that greater character pathology is associated with impairment in neuropsychological function. This relationship was present when examining all subjects together, PA and MMT subjects combined, and amongst the PA subjects only, but did not achieve statistical significance when examining the MM and non-dependent comparison groups separately.

The absence of significant findings in MM subjects is unexpected and contrary to our third hypothesis. One possible explanation for the group differences in statistical significance in the correlation of MCMI scores and neuropsychological test performance is differences between groups in MCMI scores and/or neuropsychological test performance. We have previously reported comparable degrees of personality pathology and cognitive impairment in both MM and PA subjects, with some minor variations – PA subjects tend to have more Cluster A pathology and worse visuospatial recall [8,23]. It is possible that we underestimated the importance of these minor variations, and that in fact, as Gruber et al. conclude [43], methadone treatment does selectively improve verbal and visuospatial encoding and recall in opiate dependent subjects. Such an effect could obscure the relationship between cognition and personality measures, particularly in the MM group. Similarily, Calsyn [21] reported decreases in total MCMI scores in opiate addicts after 18 months of methadone maintenance treatment. Both an improvement in neuropsychological test performance and/or a reduction in MCMI scores may result in a decrease in the correlation coefficient relating personality pathology to neuropsychological performance. The presence of methadone, possibly through the action of mediating factors, may confer a protective effect that is removed in subjects choosing to end methadone treatment. This might explain the difference in the correlation coefficient between the MM and PA subjects reported here.

We have used linear regression to examine the functional relationships of drug use history and character pathology on neuropsychological performance. In every regression model tested, the composite MCMI score accounted for a greater portion of the variance in neuropsychological performance than did any of the variables of drug use history. As a measure of the relative importance of predictor variables, the regression models suggest that drug use history has less impact on neuropsychological test performance than does personality pathology. In fact, none of the drug use history variables were any better than random chance at accounting for variance in neuropsychological scores.

Because the neuropsychological variables assess different cognitive processes, it is potentially important to distinguish which, if any, of them is most responsible for the observed relationship between neuropsychological function and personality. Interestingly, for the PA subjects only, strong negative correlations of the WAIS-R Vocabulary subscore were demonstrated with each of the MCMI Clusters scores, indicating that greater personality pathology of any sort is associated with worsened verbal function. The WAIS-R Vocabulary subscore is a test of verbal function [30], and previous research has reported decreased WAIS-R Vocabulary scores in abstinent opiate addicts compared to healthy control subjects [3]. We have shown that Stroop CW scores correlated negatively with both MCMI Cluster B and Cluster C scores in our PA subjects. This finding suggests that PA subjects who score higher in the dramatic, erratic, anxious, and fearful personality domains may have greater difficulty sustaining attention and inhibiting a habitual response. Using an emotional Stroop task to measure attentional bias, Marisssen et al [44] reported that attentional bias in abstinent opiate addicts predicted relapse to addictive drug use. Low scores on the BVRT subscore were correlated with high MCMI Cluster C scores in our PA subjects. To date, there have been no other studies of the BVRT in abstinent formerly opiate dependent subjects. In sum, these results suggest that specific neuropsychological deficits may be preferentially associated with subtypes of personality disorders. More research is needed to fully understand these correlations.

Personality disorders are thought to be enduring and stable over time [41]. Some authors have suggested that personality disorders predate substance use in opiate-addicted patients [23,45]. The data presented here points to a strong association of neuropsychological performance and a measure of personality pathology. Because of the strong association of personality pathology and neuropsychological performance, it is possible that neuropsychological deficits present in opiate dependent subjects may also be enduring and stable over time. Indeed, our previous study [8] showed no improvement in neuropsychological performance between subjects receiving methadone and those who completed a course of methadone treatment and were subsequently opiate abstinent. Similar results have been reported by Davis [46] who reported no significant difference between former opiate abusers and healthy controls on a test of verbal fluency, and Verdejo [7] who found no difference in the test performance of MM and PA subjects using a modified Controlled Oral Word Association test, the Stroop Interference test, and the Wisconsin Card Sorting test. These studies indicate that neuropsychological deficits do not necessarily resolve when drug abuse ceases.

Cognitive deficits associated with opiate dependence have traditionally been explained as arising due to drug use itself. However, based on the results reported here, we suggest that it may be more helpful to posit a multi-factorial origin to opiate-associated cognitive impairment. A variety of factors may result in neuropsychological deficits; including opiate abuse, additional concomitant illicit substance abuse, adulterants added to illicit substances, overdoses and head trauma, lifestyle and nutritional factors, infections, educational and occupational attainment, genetic and familial factors, and early-childhood experiences. It is also possible that the neuropsychological performance deficits identified in opiate-dependent subjects, like the enduring and stable personality disorders often associated with opiate dependence, may be a manifestation of a pre-existing condition that persists through drug use, abuse, and addiction treatment.

### Strengths and limitations

Insofar as possible, we have attempted to limit confounding influences on our results. We have recruited the subjects with a history of opiate-dependence from a residential treatment setting, thereby providing for a standardization of nutrition and activities, along with frequent testing for illicit substance use. We have included only MM subjects who have experienced a significant period of methadone treatment, and PA subjects with a prolonged period of drug abstinence. Nonetheless, our results must be interpreted in light of the study limitations. Firstly, this cross-sectional study can only identify associations, and does not support causal interpretations. Because entrance into abstinence treatment was entirely voluntary, it is possible that the observed differences between MM and PA groups reflect a selection bias rather than an actual effect of methadone. We have not controlled for additional past illicit drug use in our sample, and this may impact our outcome measures in unexpected ways. We have used a simple battery of cognitive tests, which limits the scope of our findings. Additionally, unmeasured factors, such as substance-related lifestyle and familial variables, may have influenced our results.

### Policy implications

Methadone has been used for years as a substitution treatment for opiate addiction [47]. Despite the amply documented success of methadone maintenance programs [48-55], there is significant public and social resistance to treating addiction with long-term prescription opiates. For their part, the patients receiving methadone also complain about the treatment and the way it is delivered. Some opponents of methadone maintenance therapy point to studies reporting opiate-induced cognitive deficits as an argument against MM treatment. In response to these concerns, Methadone-to-Abstinence treatment has received FDA approval and is considered a viable alternative to continued methadone maintenance [56]. Our study has shown no correlation between neuropsychological performance and either opiate use or methadone treatment. However, we have demonstrated a significant correlation between personality pathology and worsened cognition. Because personality is understood to be stable over time [23,24,41], we suggest cognitive deficits observed in methadone maintained patients may also be stable over time. Furthermore, there has been no published study that has established a causative link of methadone to neuronal pathology or cognitive deficits. Thus, citing opiate-induced cognitive deficits as a reason for cessation of methadone treatment makes for poor policy. There may well be good reasons to discontinue MMT in select patients. However, in the main, MMT has been shown to be effective and cost-efficient means to reduce the problems of addiction, and for the majority of patients the advantages far out-weigh purported and poorly documented disadvantages.

Current drug addiction treatment focuses on abstinence and/or harm reduction. The underlying assumption is that any existing neuropsychological and/or cognitive deficits will resolve with cessation of opiate use. If, as we have argued, neurocognitive difficulties do not correlate with opiate use, addiction treatment alone will not ameliorate impairments. Most studies show worsened treatment outcomes in addicts suffering from neurocognitive impairments [2-4,7,57-59]. Therefore, cognitive rehabilitation should be an important adjunct to drug addiction treatment. Patients entering drug addiction treatment programs should be screened for the presence of cognitive deficits. Approaches to restoring cognitive abilities and functioning can include training in goal setting and planning, developing problem-solving skills, sustaining attention, and inhibiting habitual responses. Patients will benefit most from drug addiction treatment when they can attend and receive new information and incorporate it into behavioral response [60].

## Conclusion

Subjects with a history of opiate dependence demonstrate greater levels of personality pathology than do non-dependent comparison subjects. Personality pathology is highly associated with neuropsychological performance deficits in all groups studied. The cause of this deficit is poorly understood and most likely multi-factorial. Neuropsychological deficits may not resolve with cessation of opiate abuse. Given the persistence of both personality pathology and cognitive impairment in recovering opiate addicts, cognitive remediation may help to protect against relapse.

## Competing interests

The authors declare that they have no competing interests.

## Authors' contributions

IG and EL designed the study and wrote the protocol. JP, ED, and DE participated in data analysis, interpretation, and manuscript writing. LC participated in study design and data analysis. MS participated in data collection. All authors read and approved the final manuscript.
